# Reports on the Epidemic Cholera Which Has Raged Throughout Hindostan and the Peninsula of India, Since August 1817. Published under the Authority of Government

**Published:** 1820-04-01

**Authors:** 


*Reports on the Epidemic Cholera which has raged throughout Hindostan and the Peninsula of India, since August* 1817. *Published under the Authority of Government.* One Vol. 4to. 228 Pages. Bombay, 1819.

——— seu dira per omnes

Manarent populos sævi contagia morbi.

This important series of documents, drawn up by the Medical Board of Bombay, has been presented to us, through the medium of Dr. Scott, by the desire of the head of that board, lately returned to Europe.* The work is circulating widely in India, but cannot, of course, be known here, except through such vehicle as the present. We deem it a duty, therefore, to the profession at large, to make them more intimately acquainted than they have hitherto been, with one of the most awful and fatal epidemics that ever ravaged our widely extended Indian Empire. The event itself is extremely interesting to the profession in general, in a pathological and therapeutical point of view, independently of those numerous ties and associations by which we are linked to the fate of our Asiatic possessions. On all these accounts we shall be pardoned for the length to which our analysis may extend, especially as we shall strain every nerve to make it as concentrated as literary labour and typographical closeness can render it.

There are some curious particulars attending the history of this epidemic, which are worthy of record. It first appeared in August 1817, in Zilla Jessore, about 100 miles North East of Calcutta, but without any previous peculiarity of weather; being considered by the authorities on the spot, as of a local nature, and attributable to the intemperate use of rank fish and bad rice; but it rapidly spread through the adjoining villages, running from district to district, until it had brought the whole province of Bengal under its influence. It next extended to Behar; and, having visited the principal cities West and East of the Ganges, reached the upper provinces. Through the large cities here it made a regular progress; but it was otherwise in the more thinly peopled portions of country. " The disease would sometimes take a complete circle

* Dr. Stevart, since deceased.

round a village, and leaving it untouched, pass on as if it were wholly to depart from the district. Then, after a lapse of weeks, or even months, it would suddenly return, and scarcely reappearing in the parts which had already undergone its ravages, would nearly depopulate the spot that had so lately congratulated itself on its escape. Sometimes, after running a long course on one side of the Ganges, it would, as if arrested by some unknown agent, at once stop; and taking a rapid sweep across the river, lay all waste on the opposite bank." *Report of the Calcutta Medical Board.*

In Calcutta it shewed itself in the first week of September, and each succeeding week added strength to the malady, and more extended influence to its operation. From January till the end of May it was at its acmé, during which period, the mortality in the city was seldom under 200 a week!

The centre division of the army, under the Commander in Chief, exhibited an awful specimen of the fatality of the disease. It consisted of less than ten thousand fighting men, and the deaths, within twelve days, amounted, at the very lowest estimate, to three thousand; according to others, to five, and even eight thousand!

On the 6th of August, 1818, it reached Bombay, taking about a year to cross the base of the Great Indian Delta. It appeared to Drs. Steuart and Phillips, the enlightened members of the medical board at Bombay, that the disease was capable of being " transported from place to place as in cases of ordinary contagion or infection, and also to possess the power of propagating itself by the same means that acknowledged contagions do." *Preface,* xii.

The partial and irregular manner in which the disease spread and operated in the neighbourhood of Bombay, as the cold season advanced, could not be accounted for by the medical board, " unless by supposing that a diminution of temperature, together with exposure, may have called into action some latent remains of an active poison." The board next proceeds to a description of the disease, as drawn up by the Medical Board of Bengal, which we shall here introduce verbatim.

" Having thus given a rapid and imperfect sketch of the history of the epidemic, the board should now proceed to detail the symptoms which attended its attack. This part of their task they will not find it difficult to accomplish. The leading appearances of this most fatal malady were but too well marked on their approach and subsequent progress; and amongst the myriads who were attacked, exhibited perhaps less variety and fewer discrepancies than characterise the operation of almost any other disease to which the human body is subject. The healthy and unhealthy; the strong and feeble; Europeans and Natives; the Mussulman and Hindoo; the old and young of both sexes, and of every temperament and condition, were alike within its influence.

"The attack was generally ushered in by a sense of weakness, trembling, giddiness, nausea, violent retching, vomiting and purging, of a watery, starchy, whey-coloured, or greenish fluid. These symptoms were accompanied, or quickly followed by severe cramps, generally beginning in the fingers and toes, and thence extending to the wrists and fore-arms, calves of the legs, thighs, abdomen, and lower part of the thorax. These were soon succeeded by pain, constriction, and oppression of stomach and pericardium; great sense of internal heat; inordinate thirst, and incessant calls for cold water, which was no sooner swallowed than rejected, together with a quantity of phlegm, or whitish fluid, like seethings of oatmeal. The action of the heart and arteries now nearly ceased; the pulse either became altogether imperceptible at the wrists and temples, or so weak as to give to the finger only an indistinct feeling of fluttering. The respiration was laborious and hurried, sometimes with long and frequently broken inspirations. The skin grew cold, clammy, covered with large drops of sweat; dank and disagreeable to the feel, and discoloured of a bluish, purple, or livid hue. There was great and sudden prostration of strength; anguish, and agitation. The countenance became collapsed; the eyes suffused, fixed, and glassy; or heavy, and dull; sunk in their sockets, and surrounded by dark circles; the cheeks and lips livid and bloodless; and the whole surface of the body nearly devoid of feeling. In feeble habits, where the attack was exceedingly violent, and unresisted by medicine, the scene was soon closed. The circulation and animal heat never returned; the vomiting and purging continued, with thirst and restlessness; the patient became delirious or insensible, with his eyes fixed in a vacant stare, and sunk down in the bed; the spasms increased, generally within four or five hours.

"The disease, sometimes at once, and as if it were momentarily, seized persons in perfect health; at other times, those who had been debilitated by previous bodily ailment; and individuals in the latter predicament, generally sunk under the attack. Sometimes, the stomach and bowels were disordered for some days before the attack, which would then, in a moment, come on in full force, and speedily reduce the patients to extremities.

"Such was the general appearance of the disease where it cut off the patient in its earlier stages. The primary symptoms, however, in many cases, admitted of considerable variety. Sometimes the sickness and looseness were preceded by spasms; sometimes the patient sunk at once, after passing off a small quantity of colourless fluid, by vomiting and stool. The matter vomited in the early stages was, in most cases, colourless or milky; sometimes it was green. In like manner, the dejections were usually watery and muddy; sometimes red and bloody; and in a few cases, they consisted of a greenish pulp, like half digested vegetables. In no instance was feculent matter passed in the commencement of the disease. The cramps usually began in the extremities, and thence gradually crept to the trunk; sometimes they were simultaneous in both; and sometimes the order of succession was reversed; the abdomen being first affected, and then the hands and feet. These spasms hardly amounted to general convulsion. They seemed rather affections of individual muscles, and of particular sets of fibres of those muscles, causing thrilling and quivering in the affected parts, like the flesh of crimped salmon; and firmly stiffening and contorting the toes and fingers. The patient always complained of pain across the belly, which was generally painful to the touch, and sometimes hard and drawn back towards the spine. The burning sensation in the stomach and bowels was always present; and at times extended along the cardia and œsophagus to the throat. The powers of voluntary motion were, in every instance, impaired; and the mind obscured. The patient staggered like a drunken man, or fell down like a helpless child. Head-ach over one or both eyes sometimes, but rarely occurred. The pulse, when to be felt, was generally regular, and extremely feeble, sometimes soft; not very quick; usually ranging from 80 to 100. In a few instances, it rose to 140 or 150, shortly before death. Then it was indistinct, small, feeble, and irregular. Sometimes very rapid, then slow for one or two beats. The mouth was hot and dry; the tongue parched, and deeply furred, white, yellow, red, or brown. The urine at first generally limpid, and freely passed; sometimes scanty, with such difficulty as almost to amount to strangury; and sometimes hardly secreted in any quantity, as if the kidnies had ceased to perform their office. In a few cases, the hands were tremulous; in others, the patient declared himself free from pain and uneasiness, when want of pulse, cold skin, and anxiety of features, portended speedy death. The cramp was invariably increased upon moving.

"Where the strength of the patient's constitution, or of the curative means administered, were, although inadequate wholly to subdue the disease, sufficient to resist the violence of its onset, nature made various efforts to rally; and held out strong, but fallacious promises of returning health. In such cases, the heat was sometimes wholly, at others partially restored; the chest and abdomen in the latter case becoming warm, whilst the limbs kept deadly cold. The pulse would return; grow moderate and full; the vomiting and cramps disappear; the nausea diminish, and the stools become green, pitchy, and even feculent; and with all these favourable appearances, the patient would suddenly relapse; chills, hiccup, want of sleep, and anxiety, would arise; the vomiting, oppression, and insensibility, return; and in a few hours terminate in death.

"When the disorder ran its full course, the following appearances presented themselves. What may be termed the cold stage, or the state of collapse, usually lasted from twenty-four to forty-eight hours, and was seldom of more than three complete days' duration. Throughout the first twenty-four hours, nearly all the symptoms of deadly oppression, the cold skin, feeble pulse, vomiting and purging, cramps, thirst and anguish continued undiminished. When the system shewed symptoms of revival, the vital powers began to rally; the circulation and heat to be restored; and the spasms and sickness to be considerably diminished. The warmth gradually returned; the pulse rose in strength and fullness, and then became sharp and sometimes hard. The tongue grew more deeply furred; the thirst continued, with less nausea. The stools were no longer like water; they became first brown and watery; then dark, black, and pitchy; and the bowels, during many days, continued to discharge immense loads of vitiated bile, until, with returning health, the secretions of the liver and other viscera gradually put on a natural appearance. The fever, which invariably attended this second stage of the disease, may be considered to have been rather the result of nature's effort to recover herself from the rude shock which she had sustained, than as forming any integrant and necessary part of the disorder itself. It partook much of the nature of the common bilious attacks prevalent in these latitudes. There was the hot dry skin; foul, deeply furred, dry tongue; parched mouth; sick stomach; depraved secretions, and quick variable pulse; sometimes with stupor, delirium, and other marked affections of the brain. When the disorder proved fatal after reaching this stage, the tongue, from being cream-coloured, grew brown, and sometimes dark, hard, and more deeply furred; the teeth and lips were covered with sordes; the state of the skin varied; chills, alternating with flushes of heat; the pulse became weak and tremulous; catching of the breath; great restlessness, and deep moaning succeeded; and the patient soon sunk, insensible, under the debilitating effects of frequent dark, pitchy, alvine discharges.

"Of those who died, it was believed, perhaps rather fancifully, that the bodies sooner underwent putrefaction, than those of persons dying under the ordinary circumstances of mortality. The bodies of those who had sunk in the earlier stages of the malady, exhibited hardly any unhealthy appearance. Even in them, however, it was observed, that the intestines were paler, and more distended with air, than usual; and that the abdomen, upon being laid open, emitted a peculiar offensive odour, wholly different from the usual smell of dead subjects. In the bodies of those who had lived some time after the commencement of the attack, the stomach was generally of natural appearance externally. The colour of the intestines varied from deep rose to a dark hue, according as the increased vascular action had been arterial or venous. The stomach, on being cut into, was found filled, sometimes with a transparent, a green, or dark flaky fluid. On removing this, its internal coats, in some cases, were perfectly healthy; in others, and more generally, they were crossed by streaks of a deep red, interspersed with spots of inflammation, made up of tissues of enlarged vessels. This appearance was frequently continued to the duodenum. In a very few cases, the whole internal surface of the stomach was covered with coagulable lymph; on removing which, a bloody gelatine was found laid on the interior coat, in ridges or elevated streaks. The large intestine was sometimes filled with muddy fluid, sometimes livid, with dark bile, like tar; just as the individual had died in the earlier or later periods of the attack. In most cases, the liver was enlarged, and gorged with blood. In a few, it was large, soft, light-coloured, with greyish spots, and not very turgid. In others again, it was collapsed and flaccid. The gall-bladder was, without exception, full of dark green or black bile. The spleen and thoracic viscera were, in general, healthy. The great venous vessels were usually gorged; and in one case, the left ventricle of the heart was extremely turgid. The brain was generally of natural appearance. In one or two instances, lymph was effused between its membranes, near the coronal suture, so as to cause extensive adhesions; in other cases, the sinuses, and the veins leading to them, were stuffed with very dark blood." xv.—xxi.

The following extracts will shew that the disease was known to Sydenham, and accurately described by that observant physician. He no where mentions bile as forming any part of the discharges from the stomach or bowels; and hence, it may be fairly inferred, that such discharges were not present.*

"Qui ab ingluvie ac crapula nullo temporis discrimine passim excitatur affectus, ratione symptomatum non absimilis, nec eamdem curationis methodum respuens, tamen alterius est subsellii. Malum ipsum facile cognoscitnr, adsunt enim vomitus enormes, ac pravorum humorum cum maxima difficultate et angustia per alvum dejectio; cardialgia, sitis. Pulsus celer ac frequens, cum æstu et anxietate, non raro etiam parvus et inæqualis, insuper et nausea molestissima, sudor interdum diaphoreticus, crurum et brachiorum contractura, animi deliquium, partium extremarum frigiditas, cum aliis notæ symptomatibus, quæ adtantes magnopere perterrefaciunt, atque etiam angusto viginti quatuor horarum spatio ægrum interimant."

And again, in his letter to Dr. Brady, describing the epidemics of 1674, 5, and 6, he says,

"Exeunte æstate Cholera Morbus epidemice jam sæviebat, et insueto tempestatis calore evectus, atrociora convulsionum symptomata,

---

* We have diligently searched the writings of Sydenham, and we assert, that in no one instance, when treating of cholera morbus, whether epidemic or sporadic, has he mentioned a discharge of *bile* as forming any part, much less as being the *cause* of cholera. And as Sydenham is allowed to be one of the most accurate observers of Nature, we see on what foundation Dr. Saunders and others have built their *bilious* theory of the disease. The fact is, as we have long ago stated, that the discharge of bile in cholera, is a secondary or ternary link in the chain of cause and effect—and always a sanative effort of the system, as well as a favourable symptom of the disease.

We observe too, that Areteus describes the discharge of *bile* as only an ulterior effect. "In primis, says he, quæ evomuntur, *aquæ similia sunt*; quæ anus effundit, stercorea, liquida, tetrique odoris sentiuntur. Siquidem longa cruditas id malum excitavit, quo si per clysterem eluantur, *primo pituitosa, mox biliosa feruntur.*"—*De Cholera,* Chap.5. Ed.

eaque diuturniora secum trahebat, quam mihi prius unquam videre contigerat. Neque enim solum abdomen, uti alias in hoc malo, sed universi jam corporis musculi, brachiorum crurumque præ reliquis, spasmis tentabantur dirissimis, ita ut æger e lecto subinde exiliret, si forte extenso quaquaversum corpore eorum vim posset eludere." xxiii.

The first of the foregoing extracts describes the disease with great accuracy, as it very generally affected the natives; the second is well exemplified in Dr. Burrell's Report, as it attacked the Europeans of the 65th Regiment, at Seroor. The disease is also accurately described by Girdleston, and by Mr. Curtis of Madras, in 1782, when it raged in the Southern Provinces of the Peninsula. Dr. Taylor also furnished the Medical Board with the account of a disease from a Sanscrit medical work, the MADHOW NIDAN, which clearly proves that the complaint has been long known to the natives.

" It is obviously unnecessary to prosecute this inquiry further; and we shall only add, that Dr. James Johnson is the latest author, so far as we know, who has treated this subject, and who has also the merit of having been the first who has generally pointed out the best method of cure, from a few cases he met with on the eastern coast of Ceylon, where the disease seems to be more prevalent than in any other part of India." xxviii.

The exciting and proximate causes of this interesting epidemic are, like those of most others, concealed in utter darkness—" *atra caligine mersæ*;" great discrepancy of opinion obtains in India respecting its contagious or non-contagious influence, arising naturally out of the difficulty of the subject.

" Several irresistible facts already noticed, or related in the following Reports, and its marked anomaly from all hitherto known simple epidemics, would seem to favour the doctrine of contagion, while the contrary supposition is only supported by a species of negative evidence." xxix.

The Board, however, very properly observe, that this is a question of such importance, that it ought not to be too hastily entertained as proved, nor rejected as unfounded; but prosecuted with that diligent inquiry and cautious induction, which, on every subject of science, are so necessary to the attainment of truth.

In respect to the predisposing [or rather the *exciting*] causes, practitioners are unanimous.

" Rapid atmospherical vicissitudes, in regard either to temperature or moisture; exposure of the body to currents of cold air, particularly the chill of the evening, after being heated by violent exercise of any kind, inducing debility or exhaustion; low marshy situations; flatulent or indigestible food, especially crude and watery vegetables, which compose a large proportion of the diet of the natives; and particularly that gradual undermining of the constitution which arises in a condensed, dirty, and ill-fed mass of population, are all unquestionably powerful predisposing causes."

Sad experience, however, has shewn that the absence of all these afforded no security against the attack. Whether the invisible cause (whatever that may be) acts more immediately on the vascular or nervous system, the Board cannot take upon them to determine; but from the various modes of attack which gave rise to the division of the disease into two species and varieties, they are led to the supposition that sometimes the one system, sometimes the other, bears the onus of the first onset of the malady.

" The most general attack seems to consist in a spasmodic affection of the stomach, duodenum, and more especially the biliary ducts, (the total absence of bile in the matter voided upwards and downwards being, perhaps, the most uniform characteristic of the disease) which quickly extending through the whole intestinal canal, discharges its contents. It is more than probable, however, that these are merely the first perceptible symptoms; for it would appear that a great change has already taken place in the circulating system, and that the action of the heart itself has been greatly diminished before they occur. This seems evident from the numerous cases in which neither vomiting nor purging is present, and in which the first appearance of the disease is the almost total suspension of the vital functions, immediately followed by severe spasmodic affections of the muscles and coldness of the extremities." xxxiii.

Here the Board have copied Dr. Armstrong's description of the attack of *congestive* typhus, remarking that,

" Those who are most intimate with the disease in question, will be struck with the great similarity between this and typhus, at their first appearance."

Dissections, they state, abundantly prove that venous congestion constitutes the principal change that takes place during life.

The following passage, though long, cannot be abridged, without greatly lessening its value.

" On the subject of the cure of the disease, we need say but little. The practice so judiciously and speedily adopted by Dr. Burrell in the 65th regiment, clearly proves, that at the commencement of the disease in Europeans, blood-letting is the sheet anchor of successful practice; and perhaps also with natives, provided it be had recourse to sufficiently early in the disease; and as long as the vital powers remain, so as to be able to produce a full stream, it ought perhaps never to be neglected, it having been sufficiently proved, that the great debility so much complained of is merely apparent. Calomel, as a remedy, certainly comes next in order, and when employed in proper doses, with the assistance of opium, and more particularly in the early stage of the disease, seems to be equally effectual among natives, as venesection among Europeans, in arresting its progress. In all the cases formerly alluded to, when we met the disease on its first attack, a single scruple dose of calomel, with sixty minims of laudanum, and an ounce of castor oil seven or eight hours afterwards, was sufficient to complete the cure. The practice of this place, as sufficiently appears by Dr. Taylor's report, bears ample testimony to the control which calomel possesses over the disease, in as much as it has often preserved life, when blood-letting could not be put in practice.

" All other remedies must, in our opinion, be considered as mere auxiliaries, no doubt extremely useful as such, and ought never to be neglected; but particularly the warm bath and stimulating frictions. Even where the disease appears to have given way to bleeding, we think it highly necessary constantly to administer calomel. The powerful effect of this remedy in allaying irritability of the stomach and intestines, when given in large doses, is generally acknowledged by practitioners, in the severer attacks of dysentery: as a great and permanent stimulus to the vascular system, it will be readily acknowledged by every one who has suffered for any length of time under its effect in ptyalism, where the bounding pulsations of the arteries of the temples and neck produce very disagreeable sensations, and even preclude sleep. Its powers over inflammation of the abdominal viscera, the liver in particular, and indeed, in membranous and glandular inflammation generally, are now universally acknowledged.*

---

* " We read with some surprise the declared opinion of Dr. Armstrong, in his Treatise on Puerperal Fever, that the good effects of calomel were solely owing to its purgative quality; while, at the same time, he acknowledges that the disease was both more speedily and perfectly overcome in those cases where ptyalism was produced. He has, however, made ample amends in the Treatise now before us, where he acknowledges that its value is to be attributed to its specific qualities as a mercurial. It is by the acknowledged errors of such men that medical practice is stored with its most valuable facts. Puerperal fever is not a very common occurrence in this country [India], although it occasionally takes place; and it is but an act of justice due to Dr. Helenus Scott, formerly of this place, now of Russel Square, to state, that for more than thirty years back, he was in the constant practice of treating this fever with calomel so as to affect the system; and that, to the best of our recollection, he never lost a patient. We are led to make this observation from another motive, as we have reason to believe, that this salutary practice is but little known in this country, and it may serve as a hint which may save some valuable lives."

R. STEUART,
B. PHILLIPS.

---

The

In a disease, therefore, in which we have every reason to believe that venous congestion has taken place to a great extent, and where

---

*The following Communication from Mr.* JOHN SCATCHARD, *of East Keswick, near Leeds, will come in opportunely here.*

"In the treatment of Puerperal Fever, by general professional consent, large doses of the submuriate of mercury are allowed to be of very great utility, especially when exhibited opportunely, and in concert with other well known auxiliary means. Dr. Armstrong, in his valuable work on the above disease, has very successfully employed this remedy, and thus recommends its more extensive adoption. It appears, however, from what we may collect from his work, (p. 65, 1st. edit. and p. 10, 88, 94, 2d. edit.) that neither the author, nor any of his intelligent correspondents, had recourse to this medicine, in such quantity at least, previously to January, 1813.

"Now, if your readers will take the trouble to refer to the 145th and 157th Numbers of the Medical and Physical Journal, particularly the former, it will be seen, that Dr. Bradley, of Huddersfield, administered large doses of calomel in puerperal fever, with the most unequivocal success, at least two years before Dr. Armstrong adopted the same remedy. Moreover, a reference to these cases will demonstrate, that in Dr. Bradley's hands, a combination of calomel, with opium, was beneficially employed in one instance; and in the 2d. edition of his work, Dr. Armstrong has fully canvassed this remedy. Whether the striking coincidence herein evinced were fortuitous or otherwise, it is impossible to pronounce with certainty.

"The priority of adoption and publication of the above mode of exhibiting calomel, for the cure of puerperal fever, I believe, unquestionably belongs to Dr. Bradley.

"The two papers above referred to contain the essence of almost every thing valuable that has been written on the subject of puerperal fever, since Dr. Gordon's inestimable publication on the same disease. In those papers, blood-letting and copious purging are inculcated; and more definite criteria, respecting the indications, and proper period for having recourse to the former remedy, are there pointed out, than are elsewhere to be met with. And what, I would ask, has the science of medicine, as yet, added to these means of cure, strictly speaking?

"Should Dr. Armstrong peruse these remarks, he will pardon the freedom with which they are made, and be assured that no one can have a more profound respect for his great talents than I entertain; yet, since every man is entitled to the benefit or credit of his own discoveries, I have deemed it but right unreservedly to assert the prior claim of its legitimate author to the above improved practice."

JOHN SCATCHARD.

we conclude that the liver, from its peculiar circulation and structure, is more immediately liable to become seriously and permanently injured, it should not be omitted. We have before mentioned, that Dr. James Johnson seems to have been the first who pointed out the best method of cure. Since most of the foregoing remarks were written, we have seen the second edition of that gentleman's valuable work, in which we find a strong corroborative testimony to the utility of blood-letting in this disease, or one somewhat similar to it, on the coast of Brazil, by Mr. Sheppard of Witney, without the assistance of any other remedy. The public are greatly indebted to Mr. Corbyn, of the Bengal Establishment, for his clear and comprehensive letter on this subject, at a time when the disease was producing the most dreadful ravages : the early communication of his practice has been the means of saving thousands of lives in situations where Dr. Johnson's work might not be known." xlii.

About forty official reports, from various medical officers, compose the great body of the work before us, and form the materials from which Drs. Steuart and Phillips have drawn up the foregoing luminous and interesting digest. It is not necessary to go into these reports individually. There never perhaps existed so unanimous a consent respecting the treatment of such a wide-spreading epidemic, as these documents disclose. The pre-eminent powers of blood-letting and mercury in diseases of the eastern world, are now so firmly established on the basis of facts, that it would be ingloriously to tread over a prostrate enemy, to even notice the dreams of the Brunonians, and the scepticism of the anti-mercurialists.

